# Origami Microwave Imaging Array: Metasurface Tiles on a Shape‐Morphing Surface for Reconfigurable Computational Imaging

**DOI:** 10.1002/advs.202105016

**Published:** 2022-07-27

**Authors:** Suresh Venkatesh, Daniel Sturm, Xuyang Lu, Robert J. Lang, Kaushik Sengupta

**Affiliations:** ^1^ Department of Electrical and Computer Engineering North Carolina State University Raleigh NC 27606 USA; ^2^ Department of Electrical and Computer Engineering Princeton University Princeton NJ 08544 USA; ^3^ University of Michigan‐Shanghai Jiao Tong University Joint Institute Shanghai 200240 China; ^4^ Lang Origami Altadena CA 91001 USA

**Keywords:** computational imaging, frequency diversity, metasurfaces, origami, spatial diversity

## Abstract

Origami is the art of paper folding that allows a single flat piece of paper to assume different 3D shapes depending on the fold patterns and the sequence of folding. Using the principles of origami along with computation imaging technique the authors demonstrate a versatile shape‐morphing microwave imaging array with reconfigurable field‐of‐view and scene‐adaptive imaging capability. Microwave/millimeter‐wave based array imaging systems are expected to be the workhorse for sensory perception of future autonomous intelligent systems. The imaging capability of a planar array‐based systems operating in complex scattering conditions have limited field‐of‐view and lack the ability to adaptively reconfigure resolution. To overcome this, here, deviations from planarity and isometry are allowed, and a shape‐morphing computational imaging system is demonstrated. Implemented on a reconfigurable Waterbomb origami surface with 22 active metasurface panels that radiate near‐orthogonal modes across 17–27 GHz, capability to image complex 3D objects in full details minimizing the effects of specular reflections in diffraction‐limited sparse imaging with scene adaptability, reconfigurable cross‐range resolution, and field‐of‐view is demonstrated. Such electromagnetic origami surfaces, through simultaneous surface shape‐morphing ability (potentially with shape‐shifting electronic materials) and electromagnetic field programmability, opens up new avenues for intelligent and robust sensing and imaging systems for a wide range of applications.

## Graphical abstract

Shape morphing architectures when combined with antenna/radiating elements lead to low‐profile, dynamically reconfigurable, low‐power platforms which could potentially enable joint communication‐sensing RF applications. The authors combine origami approaches along with broadband metasurfaces to demonstrate scene adaptive microwave computational imaging system. Such electromagnetic‐origami surfaces opens up new avenues in intelligent and robust sensing and imaging systems.

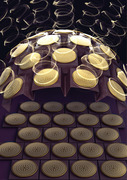

## 1 Introduction

The opening up of microwave and millimeter‐wave (mm‐Wave) part of the electromagnetic spectrum (20–100+ GHz) for fifth generation (5G) communication has to led to researchers exploiting these millimeter scale wavelengths for imaging and sensing applications for the next generation of robotics, cyber‐physical and autonomous systems, space applications, remote sensing, automotive radars, nondestructive testing, material characterization, security screening, and bio‐medical screening.^[^ ^1^, ^2^, ^3^, ^4^, ^5^, ^6^, ^7^, ^8^, ^9^, ^10^, ^11^, ^12^, ^13^, ^14^ ^]^ These waves have the ability to penetrate dielectrics, clothing, cloud, and dust, while allowing high resolution imaging due to their smaller wavelengths.^[^ ^15^ ^]^ In addition, the advancement of semiconductor chipsets operating in the microwave and mm‐Wave region, high density of integration, and high dynamic range, has resulted in scalable phased array architectures across the mm‐Wave range for communication, sensing, and imaging.^[^ ^16^, ^17^, ^18^ ^]^

Much progress has been made in computational imaging with sparse multi‐static metasurface radiators for diversity,^[^ ^1^, ^19^ ^]^ efficient broadband radiating elements^[^ ^4^, ^20^, ^21^, ^22^, ^23^, ^24^, ^25^, ^26^ ^]^ and optimization of the overall sparse array through numerical metric‐constrained optimization and physics‐based analytical approaches.^[^ ^27^, ^28^ ^]^ As a result of these physical attributes of the EM apertures, new frontier of interesting demonstrations in phased array technology for wireless communications and RF power transfer have emerged.^[^ ^29^, ^30^, ^31^, ^32^, ^33^, ^34^ ^]^ In recent years, there have been demonstrations exploiting deep neural networks, artificial intelligence, and machine learning approaches for image feature extraction and faster/improved image reconstructions.^[^ ^35^, ^36^, ^37^, ^38^, ^39^ ^]^

The properties of such imaging surfaces with respect of achievable field of view, resolution, and signal‐to‐noise ratio and depth of information, are fundamentally related to the diversity of electromagnetic fields such imaging surfaces can synthesize in frequency, space, and time.^[^ ^1^ ^]^ One constant feature of current microwave or mm‐Wave computational imaging systems is that they are predominantly planar. For most of the applications planarity is preferred due to their simplicity and ease of design and fabrication. On the other hand, it is also well established that compared to planar arrays, non‐conformal arrays provide compelling new capabilities in greatly enhancing beam scanning capabilities, field of view, allowing visual unobtrusiveness, while being compatible with the host surface on which they are deployed.^[^ ^40^, ^41^, ^42^, ^43^ ^]^ Frequency and spatial diversity are limited in passive, planar imaging arrays. Planar arrays due to the regular periodic spacing of the array elements (that defines sampling of the field), and by the 2D planar nature of its surface also suffer from limited field‐of‐view.^[^ ^44^ ^]^ Though reconfigurable EM platforms enable to overcome the diversity issue, they often also suffer from losses due the reconfigurable element in the radiating structure. The conjugation of frequency diverse electromagnetic structures and their unique arrangement on shape morphing aperture platforms could allow for interesting computational imaging modalities. Therefore, there is a significant interest in combining surface topology actuation with electronic actuations to enable unprecedented control of EM waves and open‐up new applications in sensing and imaging.^[^ ^45^, ^46^, ^47^, ^48^ ^]^

Here, we present an approach to address these particular challenges by combining for the first time origami design and folding principles for a shape‐morphing active electromagnetic surface with metasurface tiles that radiate frequency‐dependent nearly orthogonal field projections. A unique and elegant way to evaluate and build multiple shape morphing structures from a single planar surface is by exploiting the principles of origami. This allows a much richer set of radiated field synthesis rendering the ability to image complex scattering scenes with a reconfigurable field of view. At these frequencies such physical apertures are fairly large due to the their operating wavelengths and origami‐based approaches pave way to large scalable and deployable shape‐morphing structures, allowing a new direction of shape morphing, origami based sparse arrays for computational imaging.

In addition, we employ sparse computational imaging methodologies that exploit orthogonal field projections for fast image acquisition by reconstructing images from electromagnetic (EM) scattering information. Exploiting reconfigurability on both the mechanical surface (spatial diversity) and electromagnetic fields (frequency diversity), we demonstrate a programmable computational imaging system with scene‐adaptive imaging capability and reconfigurable field of view. With advances in shape‐shifting electronic materials and machine learning techniques,^[^ ^49^, ^50^ ^]^ such versatile imaging surfaces can allow adaptive and intelligent sensory interfaces for future autonomous, robotics and cyber‐physical systems.^[^ ^51^ ^]^

**Figure 1:**
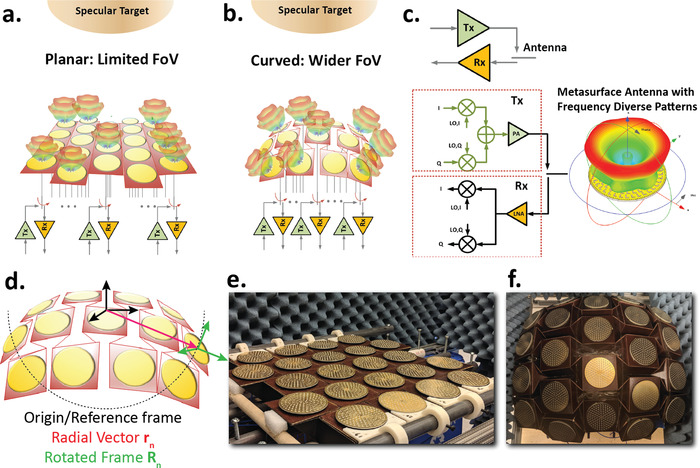
Conceptual design overview of reconfigurable origami platforms for sparse computational imaging with frequency diverse metasurfaces as antenna tiles. a) A planar array with limited field‐of‐view view where the overall imaging characteristic can be dominated by high signal‐to‐noise ratio specular reflections from the target. b) A spherically curved conformal origami surface with enhanced spatial diversity (mode orthogonality) and field‐of‐view allows synthesis of a more complex set of EM fields minimizing specular reflections, and resolving imaging details when operated in non‐planar scattering scenes. c) Quadrature transceiver architecture where any antenna tile can be reconfigured to be either a transmitter or a receiver with the ability to have complete coherent amplitude and phase detection. d) The arbitrary surface with identical radiators with its origin/reference frame at the center (black). A radial vector from the origin to a particular *n*th antenna **r**_*n*_ is shown in red and the antenna's corresponding rotated frame (green) which in turn represents the associated rotation matrix *R*_*n*_ is shown in green. e,f) The shape reconfigurable grafted Waterbomb origami structure with 22 metasurface antenna tiles radiating orbital angular momentum (OAM) modes in the K‐band (17‐27 GHz). These antennas in combination with reconfigurable origami structure exhibit both spatial and frequency diversity.

The range of reconfigurable shapes determine the versatility of the imaging surface. This influences the choice of the folding patterns that needs to be co‐designed with the electromagnetic tiles to achieve the desirable electromagnetic field diversity. The conceptual design of such a reconfigurable origami platform with multi‐static metasurface antenna transceiver tiles is shown in **Figure** **1**a– c. As shown in the figure, a planar array has a limited field‐of‐view and image reconstruction is affected by the high specular reflections from the target. In comparison, a spherically curved conformal origami surface can allow enhanced spatial diversity (mode orthogonality) and field‐of‐view while minimizing specular reflections. Each active EM tile can be interfaced with a quadrature transceiver architecture, allowing either coherent excitation or reception with both amplitude and phase information.

A computational imaging system relies heavily on the image transfer matrix that electromagnetically relates the unknown scattering object and the physical complex measurement vector.^[^ ^52^ ^]^ For an effective image reconstruction, the image transfer matrix has to be as orthogonal as possible such that the principal component analysis leads to a hypersphere.^[^ ^53^ ^]^ In reality, this may not be ideally achievable. However, one could approach more closely to a hypersphere by designing a radiating aperture that exploits spatial and frequency diversity. Spatial diversity could be achieved by a reconfigurable origami platform, while frequency diversity is usually a feature of the radiating element. Polarimetric imaging,^[^ ^54^ ^]^ on the other hand, can be achieved by using radiators with two orthogonal polarizations or through sophisticated shape morphing platforms which have the ability to rotate individual tiles about an axis. The generalized far‐field pattern of such an arbitrarily oriented sparse array with identical radiators is given by^[^ ^55^ ^]^

(1) $$F\left(\hat{r}\right)=\sum_{n}a_{n}\;e^{j\beta \hat{r}\cdot r_{n}}\;R_{n}\;f\left(R_{n}^{-1}\hat{r}\right)$$

where β is the propagation constant, $f(\hat{r})$ is the far field antenna pattern which is solely the property of the electromagnetic radiator, *a*_*n*_ is the associated complex amplitude of the *n*th antenna tile, **r**_*n*_ is the position of the *n*th antenna tile in some reference frame, *R*_*n*_ is the associated rotation matrix of the *n*th antenna tile. The shape of the underlying structure and in turn the position of the antenna tiles are governed and described by **r**_*n*_ and *R*_*n*_ that represent the geometrical characteristics of the overall surface. Thus the radiating element is solely responsible for the frequency diverse nature of the far‐field pattern^[^ ^3^ ^]^ while the underlying arbitrary surface (which could potentially be reconfigurable) provides spatial diversity. Figure 1d pictorially represents the position vector and the associated rotation matrix of a radiator element, **r**_*n*_ and *R*_*n*_, respectively, in such an arbitrarily curved topology. Figure 1e,f shows the fabricated reconfigurable origami platform with 22 identical metasurface antennas on each tile.

The technological tools for fabricating high‐frequency electromagnetics are, by and large, oriented around planar fabrication. Achieving spatial diversity that exceeds what is achievable with complete control of each antenna element of planar array requires deviations from planarity and isometry, through: spreading, curving, and stretching of the placement of antenna elements. Given that individual elements are typically rigid, a spatially diverse architecture inclines the architecture toward a system consisting of rigid panels joined by deformable regions.

When those deformable regions take the form of hinges, the structural mechanism—panels connected by hinges—can be viewed as a foldable surface, and this, in turn, allows us to tap into the rich field of folding mechanisms associated with origami, the art of paper folding. Many of the structures of origami, along with their associated mathematical design principles, naturally lend themselves to the realization of reconfigurable, conformable, and deployable surfaces. Such shape‐changing morphological structures have also found applications in antenna design,^[^ ^56^, ^57^ ^]^ filters,^[^ ^58^ ^]^ molecular self‐assembly,^[^ ^59^ ^]^ solar panel deployment in spacecraft,^[^ ^60^, ^61^, ^62^ ^]^ biomedical devices like vascular stents,^[^ ^63^ ^]^ and consumer products such as umbrella, lampshade, canoe, water bottles, and so on.^[^ ^64^ ^]^ Such reconfigurable surfaces could potentially be also used as imaging apertures which exploit spatial diversity. In the past decade, various mechanisms of such control on origami surfaces have been demonstrated including mechanical actuation,^[^ ^65^, ^66^ ^]^ thermal triggering,^[^ ^67^ ^]^ magnetic field control,^[^ ^68^, ^69^ ^]^ pneumatic control,^[^ ^70^ ^]^ fluidic actuation,^[^ ^71^ ^]^ and with actuable materials such as shape memory alloys.^[^ ^72^ ^]^

With this motivation, we employ a reconfigurable origami platform with embedded frequency diverse metasurface antennas to perform scene adaptive, sparse, and microwave computational imaging. The aperture can be reconfigured for a specific imaging metric, namely, the signal‐to‐noise ratio of the measurements, cross‐range resolution, or field‐of‐view. Mechanical self‐reconfigurability can be enabled though multiple actuations means depending on the exact nature of application. This combination of active EM surfaces with signal processing ability in each panel opens up new directions for future multi‐functional, large‐scale, adaptive, and low‐cost mm‐wave imaging and sensing systems for autonomous vehicles, robotics, and cyber‐physical systems.

The article is structured as follows. First, we discuss the design and implementation of a specific origami based shape morphing aperture which acts as the structural platform for radiating elements. Then we discuss in detail the design and characterization of frequency diverse metasurface antenna followed by the computational design framework. Last, we design and characterize scene‐adaptive computational imaging experiments followed by the respective experimental validation of both 2D and 3D stand‐off image reconstructions.

## 2 Origami Design Principle for Spatial Diversity and Field of View

To allow for a surface that has a broad range of conformal structural variability for imaging and to mount rigid planar antennas, several origami design constraints needs to be addressed. The origami design has to be scalable in its architecture with rigid panels (antenna tile) and flexible hinges with the fabrication process being planar with individual panels (for mounting antennas) with a relatively large fill factor. Finally, the structure has to support multiple stable deformation modes, namely, planar, cylindrical along the *x*‐axis, cylindrical along the *y*‐axis, and spherical segment.

The requirement for rigid panels meant that the range of possible fold patterns is limited to this with *rigid foldability* (which is a relatively small subset of all possible folding patterns). Achieving the first three deformation forms—planar, *x*‐cylinder, *y*‐cylinder—is relatively straightforward, since all three are developable surfaces, and thus are isometric with one another. However, a spherical surface has positive Gaussian curvature, and thus requires a fold pattern whose low‐energy deformation modes include such a surface.

**Figure 2:**
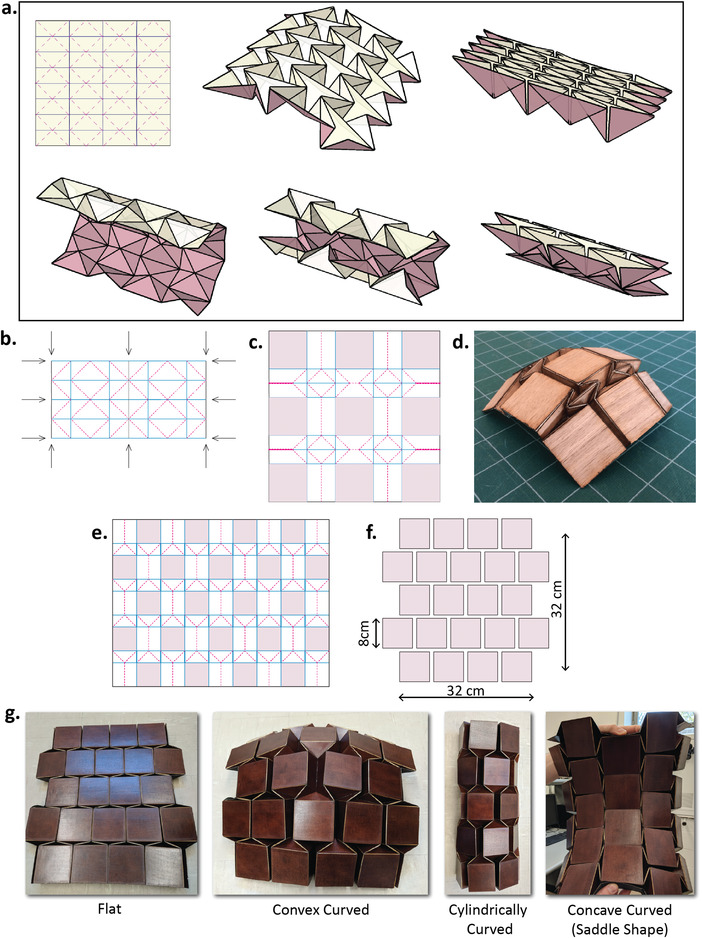
Origami surface design for the reconfigurable conformal computational imaging array. a) Cylindrical deformation modes of the Waterbomb tessellation pattern. The figure shows the crease pattern, lateral cylindrical curvature, and top‐to‐bottom cylindrical curvature of the same pattern. b) Crease pattern for a portion of the Waterbomb tessellation. Arrows indicate where square grafts are inserted. c) The grafted pattern. The inserted square tiles are shaded with an individual tile dimension of 8 cm × 8 cm. d) A folded example in spherical deformation mode. e) Crease pattern of the offset‐panel tessellation. f) Arrangement of square tiles in the flat state of the folded form. g) Fabricated reconfigurable grafted Waterbomb origami platform showing flat, convex, cylindrical, and concave curved shapes.

A well‐known folding pattern that has a spherical deformation mode is the Waterbomb tessellation, (ref. [73]; pp. 169–173) this pattern can be deformed in all three desired modes with low deformation energy. **Figure** **2**a shows the two cylindrical deformation modes of this pattern. However, the panels of the pattern are all nearly orthogonal to the desired cylindrical or spherical surface. It is possible, though, to insert square tiles into the pattern, using the well‐known folding design technique called *grafting*.^[^ ^74^ ^]^ This gives rise to a fold pattern that, in its fully folded form, realizes an *n* × *N* array of square tiles, with the individual tiles conformal to the desired surface: planar, cylindrical, or spherical.

Figure 2b,c shows the progression from the basic Waterbomb tessellation, to a grafted form, and a spherically deformed folded physical model (Figure 2d). This pattern exhibits all of the desired deformation modes, but a relatively large fraction of the initial surface goes into the portion of the fold pattern that gave the desired kinematics. It can be noted that a second set of grafted tiles can be inserted into the pattern in such a way as to achieve the same range of deformable surfaces with fewer folds per tile, simply by allowing adjacent columns of tiles to be offset. The result, shown in Figure 2e,f, has half as much material between the panels (as can be seen by the amount of white between the shaded square panels) but still provides the deformation modes attainable from the underlying Waterbomb tessellation.

While we achieved this pattern by evolution from the initial Waterbomb pattern, like many periodic folding patterns, there are other structures that shape this can evolve from as well.^[^ ^75^ ^]^ As illustrated in Figure 2, this pattern fabricated on a 4/5/4/5/4 array, allows us to explore the shape reconfigurable imaging array with spatial, frequency, and field diversity.

## 3 Metasurface Antenna with Frequency Diversity: Design and Characterization

In order to exploit frequency diversity, a broadband, high efficiency, leaky‐wave cavity‐backed metasurface antenna is chosen as a transceiver front‐end of the imaging system. The metasurface antenna consists of “L” shaped sub‐wavelength leaky wave slots on the top metal layer. The leaky slots are arranged in a radially periodic manner preserving radial symmetry. The bottom layer is the ground plane and the two metal layers are separated by a Rogers 4003 low‐loss dielectric substrate of thickness 1.52 mm. The top and bottom layers are connected through a metallic via, thus forming a complete cavity‐backed structure. These vias are essential to form a closed cavity in order to ensure that most of the energy inside the cavity is radiated out through the sub‐wavelength leaky structures. In this metasurface antenna structure most of the energy is radiated through the slots well before the energy reaches down to the via edge. The cavity ensures a guided mode inside it and the via‐cage around the cavity ensures minimal coupling between the metasurfaces (see Figure S11, Supporting Information). The radiation of the antenna does not necessarily rely on the quality factor of the cavity. The sub‐wavelength “L” shaped leaky slots are arranged periodically in a radial manner and the number of slots scales up proportionally with the ring number, *n*. The total number of slot array rings are six in a given radius of 7 cm with a total number of slots given by, $\sum_{n=1}^{6}6\;n\;=\;126$. The metasurface antenna is fed centrally through a tab‐contact coaxial Sub‐Miniature version‐A (SMA) connector. This coaxial feed is used to launch radially propagating TM01 mode inside the cavity^[^ ^76^ ^]^ (as shown in Figure S1, Supporting Information). The response of the antenna changes dramatically at different frequencies as the radially propagating mode interacts differently with the sub‐wavelength slot arrays, thus generating frequency diverse far‐field patterns.

The metasurface antenna design dimensions and the fabricated structure are shown in Figure S2a,b, Supporting Information, respectively. The shape reconfigurable origami platform consists of 22 titles and one metasurface antenna mounted on each tile. The metasurface antenna was designed and simulated using commercial electromagnetic software and was optimized to operate in the K‐band frequency range (18–27 GHz). The simulated and measured absolute reflection coefficients (*S*_11_ magnitude) are shown in Figure S2c, Supporting Information. The *S*_11_ measurements are repeated for all the 22 antennas used in the setup and all the antennas exhibit similar performance with an overall measurement variation in *S*_11_ of ≈±0.5 dB showing good repeatability across the antenna arrays. We also ensure that the S‐parameters of the switch matrix network (shown in Figure S12, Supporting Information) is very well embedded and calibrated across the array. The antenna radiates with average radiation and total efficiency of 80% and 50%, respectively, as shown in Figure S2d, Supporting Information.

**Figure 3:**
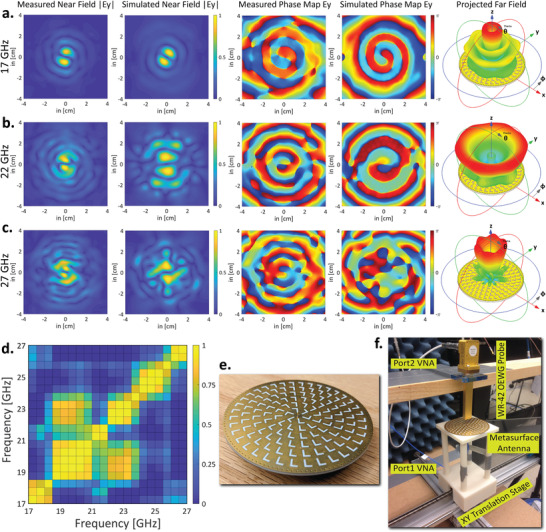
a–c) The simulated and measured spatial near‐field ***E*_*y*_** norm and their phase profiles at three distinct frequencies, namely, 17, 22, and 27 GHz. Corresponding far‐field patterns demonstrate the frequency diverse characteristics of the designed metasurface antenna. The spatial near‐field ***E*_*x*_** component is not plotted since its exactly orthogonal to ***E*_*y*_** component due to the radial symmetry of the antenna design. d) The correlation between far field patterns across discrete frequencies between 17–27 GHz demonstrating the frequency diverse nature of the metasurface antenna. e) Shows the fabricated metasurface antenna. f) Near field scan setup showing the metasurface antenna on a xy‐scanner stage and the WR‐42 open‐ended waveguide near field probe.

The metasurface antenna is further characterized by performing near‐field scans using open‐ended waveguide (OEW) WR‐42 probes. The near‐field scans are performed across the operating band (17–27 GHz) with incremental frequency steps of 0.1 GHz (101 points) and these vector fields are later used to build the forward model image transfer matrix used in computational imaging experiments. The measurement setup of the near‐field characterization is explained further in the Experimental Section. The measured electric field norms and phase maps at three distinct frequencies (17 GHz (lower), 22 GHz (mid), and 27 GHz (higher)) are shown in **Figure** **3**a– c, respectively. In order to quantify the frequency diverse nature of the metasurface antenna, we analyze the correlation between the far‐field patterns across discrete frequencies. The correlation matrix of the far‐field patterns is shown in Figure 3d. The correlation matrix of the far‐field patterns acts as a metric to demonstrate how a radiating element far‐field patterns change drastically over the frequency range. The sparse, block‐diagonal nature of the correlation matrix demonstrates the frequency diverse characteristics of the metasurface antenna while maintaining the overall radiation efficiency above 80% across the working frequency range (see Figure S2, Supporting Information). The measurement setup showing the custom near‐field measurement setup with OEW WR‐42 probe and the metasurface antenna (also shown in Figure 3e) on the xy‐scanner is shown in Figure 3f.

The near‐field patterns and phase profile at lower band clearly show a $L_{1}^{|-1|}$ Laguerre– Gaussian orbital angular momentum (OAM) mode, thus creating a conical, cylindrically symmetric far‐field pattern with a null at the broadside.^[^ ^77^, ^78^, ^79^ ^]^ The generalized Laguerre– Gaussian (LG) beam propagating in the *z*‐direction is of the form^[^ ^80^ ^]^

(2) $$\begin{array}{ccc} E_{p,\ell }\left(\rho ,\phi ,z\right) & = & E_{0}\frac{w_{0}}{w(z)}\;e^{i\ell \phi }{\left(\frac{\rho }{w(z)}\right)}^{|\ell |}L_{p}^{|\ell |}\left(\frac{2\rho^{2}}{w^{2}\left(z\right)}\right)\exp\left(\frac{-\rho^{2}}{w^{2}\left(z\right)}\right)\\ & & \times \exp\left(-i\beta z-i\frac{\beta \rho^{2}}{2R(z)}+i\Psi_{p,\ell }\left(z\right)\right) \end{array}$$

where *p* is radial mode number, ℓ is the angular mode number, *z*_0_ is the Rayleigh range, $w\left(z\right)=w_{0}\sqrt{1+\frac{z^{2}}{z_{0}^{2}}}$ is the spot radius as the wave propagates, $R\left(z\right)=z\left(1+\frac{z_{0}^{2}}{z^{2}}\right)$ is the wavefront curvature, $L_{p}^{|\ell |}$ is the associated Laguerre polynomial, and $\Psi_{p,\ell }\left(z\right)=\left(|\ell |+2p+1\right)\;\mathrm{atan}\left(\frac{z}{z_{0}}\right)$ is the phase function. The phase profile evolution of a $L_{1}^{|-1|}$ mode Laguerre– Gaussian (LG) beam along the *z*‐direction is shown in Figure S3, Supporting Information. At the higher band, the phase profile indicates a combination of multiple OAM modes. This can be further explored by either decomposing the fields into respective LG OAM modes or cylindrical harmonic modes.^[^ ^76^ ^]^ Both these techniques form a complete orthogonal basis set. The cylindrical harmonic decomposition technique is described in Section S1, Supporting Information.

## 4 Computational Imaging System with Metasurface tiles on Shape‐Morphing Origami Surface: Design and Measurement

The origami platform based imaging system consists of 22 metasurface antenna tiles, of which 11 are preselected transmitters (Txs) and receivers (Rxs) only. The antennas are connected to a high‐speed RF electro‐mechanical switch matrix which in turn connects to any two given antennas in the system to a 2‐port vector network analyzer (VNA). Port 1 of the VNA acts as a swept frequency signal source and Port 2 as the receiver back‐end. The preselected Tx and Rx antenna tile set on the origami and the switch matrix setup are shown in Figure S12, Supporting Information.

In this imaging system, first‐order Born approximation is assumed to represent the target objects with regularly spaced grid point scatterers.^[^ ^81^ ^]^ The complex‐valued measurement vector, **g** and the complex‐valued scattering coefficients of the target, **f** are related linearly through an image transfer matrix **H**. This is often called the forward model and is represented by the equation,

(3) $$\begin{array}{c} g_{M\times 1}=H_{M\times N}\;f_{N\times 1} \end{array}$$

where *M* is the total number of complex‐valued measurements and *N* is the total number of scattering grid points or voxels in the scene to be imaged. The forward model or the image transfer matrix, **H** is constructed apriori by measuring or calculating the propagated vector fields at the required scene volume. In this regard, to precisely construct the forward model, we perform near‐field scans of the metasurface antenna across frequency, convert these fields to dipole moments using surface equivalence theorem (Schelkunoff's equivalence), then individually propagate these vector dipole moments using Green's function to a scene voxel, and sum all the responses from all the dipole moments for a given voxel.^[^ ^4^, ^82^ ^]^ Further details of this method are provided in Section S1, Supporting Information. This procedure is repeated for all the voxels in the scene volume and this approach is computationally efficient and easily parallelizable compared to other Fourier transform techniques for a given limited computational resource. However, computationally accurate and fast Fourier accelerated have been recently demonstrated which provides comparable performance to that of Green's function approach.^[^ ^83^ ^]^ The elements of matrix **H**_*ij*_ are proportional to $E_{i}^{\mathrm{Tx}}\cdot E_{j}^{\mathrm{Rx}}$, where $E_{i}^{\mathrm{Tx}}$ is the *i*th transmitter and $E_{j}^{\mathrm{Rx}}$ is the *j*th receiver in the imaging aperture plane. The proportionality constant includes non‐free space parts of the signal path and their associated calibration coefficients.^[^ ^82^ ^]^ The total number of measurements, *M* = *N*_Tx_ × *N*_Rx_ × *N*_f_ = 11 × 11 × 101 = 12221, where *N*_Tx_ and *N*_Rx_ are the total number of transmitters and receivers, respectively, in the aperture plane and *N*_f_ is the total number of frequency points. In order to reconstruct the under‐sampled scenes (**H** is usually underdetermined), we employ regularized least squares approach to estimate the scene vector **f** and the estimate **f**_est_ is found by solving *argmin*_*f*_(‖**g** − **Hf**‖_2_ + Γ‖**f**‖_1_) that minimizes this expression, where Γ is the regularization coefficient.^[^ ^82^, ^84^, ^85^ ^]^

### 4.1 Reconfigurable Cross Range Resolution with Stretchable Origami

**Figure 4:**
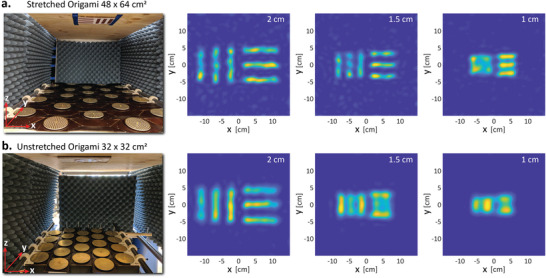
Stand‐off 2D and 3D scattering target image reconstructions for different origami platform shapes. a,b) The 2D image reconstructions of cross‐range resolution targets at a stand‐off of 1 m. In the stretched origami case, the aperture area is 48 × 64 cm^2^ and achieves a cross‐range resolution of 1 cm in the *y*‐direction. In the unstretched origami case, the aperture area is 32 × 32 cm^2^ and achieves a cross‐range resolution of 2 cm. Planar apertures easily capture specular reflections from planar targets. Reconfigurable origami apertures provide a control to adaptively change the cross‐range resolution of the images.

The ability of the origami surface to adaptively stretch and increase its aperture can allow the imaging surface to intelligently optimize its resolution. We perform such a computational imaging experiment to illustrate this capability. The cross‐range resolution of a coherent imaging system is given by $\delta_{\mathrm{cr}}=\frac{\lambda r_{0}}{2D}$, where λ is the operating wavelength, *r*_0_ is the stand‐off distance between the aperture plane and the imaging/scene plane, and *D* is the effective size of the aperture (largest baseline between any Tx and Rx pair.) The origami aperture is reconfigured to two extreme cases, that is, stretched and unstretched cases to determine the minimum and maximum achievable cross‐range resolution using standard targets of 2, 1.5, and 1 cm. In the stretched case the origami assumes an aperture area of 48 × 64 cm^2^ as shown in **Figure** **4**a. Due to increase in effective aperture area along *y*‐direction, this in turn leads to the best resolution of 1 cm in the *y*‐direction. As can be seen, both 2 and 1.5 cm resolution targets are well resolved in both the directions and can be seen in the reconstructed images. In the unstretched case, the origami assumes an aperture area of 32 × 32 cm^2^ as shown in Figure 4b. This leads to the best resolution of 2 cm in both *x*‐ and *y*‐directions. However, as can be seen, 1.5 and 1 cm targets are not resolved. It can be noted that the images in the stretched case are relatively noisier than the unstretched case, since the latter spans high **k** vectors that often lead to low signal to noise measurements.^[^ ^86^, ^87^ ^]^ These high **k** vectors are responsible to extract the scene information with stretched apertures that lead to a better resolution. The measured and predicted cross‐range resolutions are fairly accurate for a mid‐band wavelength of λ_mid_ = 1.3 cm corresponding to 22 GHz. Such planar aperture configurations are ideal for planar specular targets and the reconfigurable nature of the origami platform helps to adaptively change the cross‐range resolution metric of the reconstructed images.

### 4.2 Reconfigurable Field of View and 3D Imaging with Curved Origami Surface

**Figure 5:**
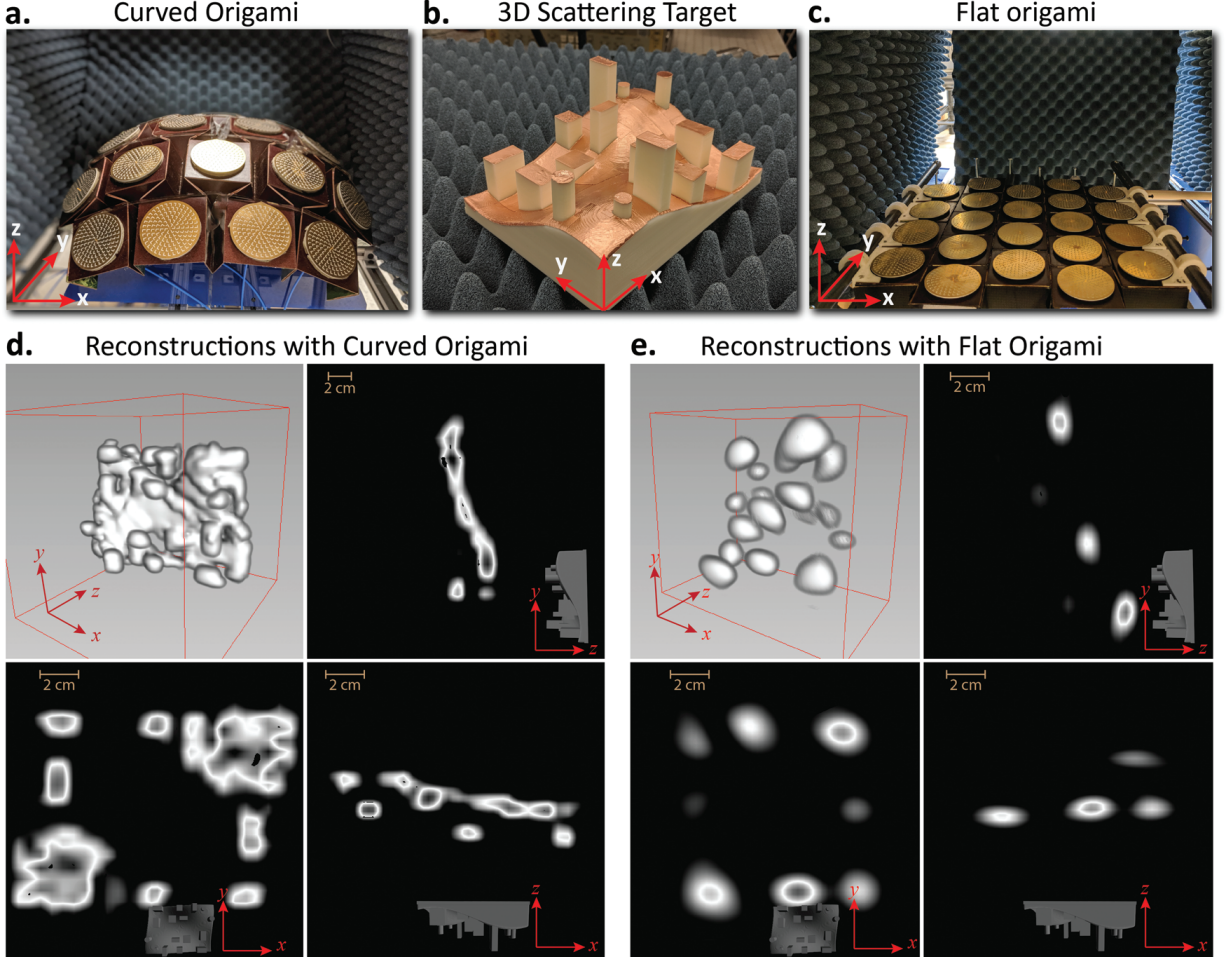
Stand‐off 3D scattering target image reconstructions for different origami platform shapes. a) The origami platform set in its spherically curved morphology state. b) The scattering 3D target is shown. c) The origami platform set in its flat planar unstretched morphology state. The target consists of cross‐range widths/lengths and depth‐range heights varying from 2 to 1 cm. d,e) The combined image reconstructions of the 3D scattering target at a stand‐off distance of 1 m using curved and flat origami morphology, respectively. These figures include the perspective view and different cut plane views comparing the image reconstructions of the 3D target using different morphology. The shape reconfigurable origami demonstrates the ability to adaptively change the field‐of‐view and demonstrate detailed 3D structures of the scene due to the diversity of the EM fields radiated in space, when compared to highly specularly dominated images obtained in the planar imaging array configuration.

The shape morphing property of the origami can allow the surface to adjust each antenna orientation, change the overall surface topology, and fundamentally synthesize different electromagnetic fields in space. Together with this spatial diversity from the topology and frequency diversity from antenna elements, the origami imaging array can adaptively reconfigure its field‐of‐view of the overall imaging aperture, including the ability to image complex objects and overcoming high specular reflections. We compare the 3D imaging performance of the reconfigurable origami in its two states, namely, the curved spherical (as shown in **Figure** **5**a) and flat planar state (as shown in Figure 5c).

The depth resolution of a transceiver based imaging system is given $\delta_{\mathrm{dr}}=\frac{c}{2B}$, where *B* is the total bandwidth. To demonstrate the ability of reconstruction 3D objects, we 3D printed a complex scattering object with varying widths, lengths, depths ranging from 2.5 to 1 cm. The 3D printed structure is shown in Figure 5b. The transverse size of the target object is ≈18 cm × 13 cm. The image reconstructions were performed for spherically curved and flat origami configurations as shown in Figure 5a,c, respectively. The image reconstruction of the complex 3D target using curved origami is compared with the ground truth and the comparison is shown in Figure S5, Supporting Information. This particular experiment aims to elucidate the ability to control the field‐of‐view of the overall imaging aperture by curving the origami structure. The 3D printed target with metallic top surface is symmetric about the diagonal with undulating surface and variable projection stubs sticking out of the surface which help to characterize both the complex specular reflections and depth resolution of the reconstructed image simultaneously. In both the origami configurations, three image reconstructions were performed with the scattering object at the center, 0.5 m left offset, and 0.5 m right offset with respect to the aperture. Later the three image reconstructions were combined accordingly and thresholded to form the final reconstructions. Though we show reconstructions for a spherically curved origami structure, the same approach can be used to perform imaging with concave and cylindrical origami structures. The current Waterbomb origami platform allows for these deformations. Such configurations could bring in different scope and enhancements toward the imaging properties including focusing of electromagnetic energy and minimizing the effect of specular reflections. However, slight modifications are required in the current setup to accommodate antennas on the origami platform in an inward‐cylindrical or concave fashion. Figure S6, Supporting Information, also shows a comparative image reconstruction of a simple cylindrical target with the curved and planar origami platforms (including both stretched and unstretched cases).

To allow the forward model computation for sparse imaging, the location and the orientations of the different antennas in the curved origami structure were determined through structured light measurement using a commercial off‐the‐shelf 3D scanning system. The 3D image stereographic camera images of the flat and curved origami are shown in Figure S7, Supporting Information, and can be processed to determine the center locations of the antenna tiles relative to a reference point. The 3D video rendering of the reconstructions was performed using ImageVis3D software^[^ ^88^ ^]^ and the videos are provided in Supporting Information. Further details of this particular experimental setup are shown in Figure S8, Supporting Information. When the origami is reconfigured between two states, the effect of misalignment on image reconstructions have not been studied in this article. However, the origami structure ensures highly stable transitions between two states, as intermediate states are not permissible in such structures. There are algorithmic approaches to correct for any minor misalignment errors.^[^ ^89^, ^90^ ^]^ Another potential approach to correct for misalignment errors is through smart actuator mechanism on origami structures which could adaptively correct the surface topology based on the feedback from the distributed stereographic sensors or by the stand‐off image reconstruction.

In the curved origami configurations, the image reconstructions demonstrate detailed 3D structures of the scene due to the diversity of the EM fields radiated in space and improved field‐of‐view, when compared to highly specularly dominated images obtained in the planar imaging array configuration. The perspective view and different cut plane views of the reconstructed final image for the curved origami configuration and the planar configuration are shown in Figure 5d,e, respectively. As can be seen from the figures, the flat imaging array severely underperforms due to high specular reflections, where most of the details of the complex scene get hidden. The curved spherical topology, on the other hand, through the synthesis and reception of a much richer set of *k*‐vectors resolve the details that get buried due to specular reflections. The total number of voxels estimated in each image reconstructions were about *N* = 117 740 which is ≈9.6 times the number of measurements (total number measurements *M* = 12 221). This, in turn, makes the **H** matrix highly under‐determined, thus requiring sparse computational frequency diverse techniques to perform image reconstructions. The choice of the frequency step is a critical parameter that affects the under‐determine factor of the image transfer matrix. Thus a frequency step of 0.1 GHz is chosen to ensure a good balance in this factor.

**Figure 6:**
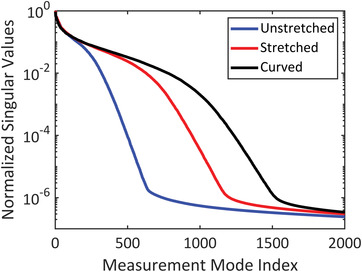
Principal component analysis (PCA) of the image transfer matrix **H** for different origami structures.

In order to mathematically access the performance of different origami configurations, we perform principal component analysis (PCA) of the image transfer matrix, **H** as shown in **Figure** **6**. Clearly, the curved origami structure outperforms in terms of orthogonality metric which a key metric for computational imaging systems.^[^ ^24^, ^91^ ^]^ The orthogonality and conditionality of image transfer matrix **H** which relates the physical measurements **g** and the unknown scene voxel matrix **f** is the key to such computational imaging systems. In order to analyze the orthogonality metric of **H** matrix we perform singular value decomposition of this matrix for different origami platforms. As shown in Figure 6, the slope of the unstretched origami is much sharper compared to the spherically curved origami. The combination of frequency diverse metasurface antennas and the unique arrangement of these radiators on the reconfigurable origami shapes indeed enhance the orthogonality metric of the image transfer matrix. In all of the three cases, we ensure that the target or the scene is 1 m away from the topmost surface of the multi‐static imaging aperture plane. However, it should be noted that there is trade‐off between the orthogonality and signal‐to‐noise ratio of the physical measurements.^[^ ^82^ ^]^ Using Shannon– Hartley theorem and standard error propagation, one can relate the singular values of **H** matrix and measurement noise δ*g* to determine the total measurement–added information as follows

(4) $$\begin{array}{c} Q=\sum_{m=1}^{M}Q_{m}=\Delta tB\sum_{m=1}^{M}\log_{2}\left[{\left(\frac{\Delta f}{\delta g}S_{mm}\right)}^{2}+1\right] \end{array}$$

where Δ*tB* is the measurement time–bandwidth product, δ*g* is the measurement noise, Δ*f* is the spread of scattering amplitudes over the scene ensemble **f**, and *S*_*mm*_ is the *m*th singular value of the image transfer matrix **H**.

The ability to reconfigure the overall aperture thus allows an adaptive control over critical imaging metrics such as spatial diversity, cross‐range resolution, field‐of‐view, and signal‐to‐noise ratio. Such origami structures can be provided with electronic actuation to allow for electronic shape morphing and dynamic imaging optimization. While we demonstrate such diversity with three surface conformations, namely, the curved, flat‐stretched, and flat‐unstretched platforms, more complex shapes can be achievable with other forms of origami folding patterns that can allow targeted optimization of other imaging functionalities of interest. Being able to achieve high resolution imaging against complex scenes in a robust and repeatable fashion is critical for sensory perceptions of autonomous systems, and such shape‐shifting, active EM surfaces can enable such modalities.

## 5 Conclusion and Future Outlook

The ability to simultaneously program the conformation of the EM surface and the EM field configurations can significantly advance mm‐wave sensing and imaging. As we demonstrate here, through origami actuations, the imaging surface described here, allows a nearly 2× enhancement in lateral resolution, and reconfiguration of field of view to allow capturing of complex reflecting scenes. In addition, being able to synthesize and sense with high sensitivity across a broad frequency range (17–27 GHz) can allow hyper‐spectral imaging, and dynamic optimization of both high lateral and depth resolution. To combine effectively information from diversity of EM fields for robust operation complex scattering scenes, sensor fusion algorithms are becoming increasingly attractive. When combined with advancements in machine learning, convolutional neural networks (CNNS), such systems can lead to intelligent and adaptive imaging systems deployable in a wide range of applications.

The approach of combining programmable surface topology *with mechanical actuations* and reconfigurable EM field distribution *with electronic actuations* (such as with orthogonal frequency diverse fields in this work) opens up a completely new design space for versatile imaging arrays. Dynamic co‐optimization of both of these aspects can result in new design strategies, algorithms, image acquisition methodologies and a different trade‐off space.^[^ ^28^ ^]^ Here, we demonstrate one possible configuration with a modified Waterbomb origami surface that can mount rigid metasurface EM tiles on panels connected with flexible hinges supporting multiple deformation stable modes. In particular, we exploit surface topology modification across unstretched, stretched, and spherically curved surfaces, and combine with near‐orthogonal modes from the metasurface panels across 17–27 GHz to demonstrate both 2D and 3D diffraction‐limited image reconstructions with adaptive imaging metrics in terms of signal‐to‐noise ratio, cross‐range resolution, and field‐of‐view. The paper also presents the fundamental techniques that describe the method of optimal origami design for spatial diversity, the design of the electromagnetic surface radiating orthogonal fields for frequency diversity, and the associated signal processing algorithms for image construction.

The applications of shape morphing surfaces to realize reconfigurable EM structures are getting increasingly popular for antennas, solar panels, and in applications where tuning and compactness in folding are critically important. While realized on wooden panels, the proposed system can certainly be replaced by lighted weight materials allowing for large‐scale deployment in autonomous vehicles, robotic systems, and spacecraft. There are on‐going efforts to realize shape morphing origami structures with programmable hinges, shape memory alloys, and liquid crystal elastomers.^[^ ^32^, ^58^, ^92^, ^93^, ^94^, ^95^ ^]^ By eliminating the constraints of planarity and isometry, we demonstrate the ability to combine origami principles, high‐frequency electronics, and signal processing to realize versatile imaging and radar systems. The presented approach can pave the way for future multi‐functional, large‐scale, adaptive, and low‐cost microwave and mm‐Wave imaging and sensing systems for autonomous vehicles, robotics, and cyber‐physical systems. When combined with shape morphing electronics (with shape memory allows, liquid crystal elastomers) and silicon‐based high‐frequency electronics, such approaches can foster a new class of low‐cost, scalable, lightweight, and intelligent sensing and imaging systems.

## 6 Experimental Section

### Origami Fabrication

Fabrication of the flexible origami structure was accomplished using a previously described^[^ ^96^ ^]^ self‐aligned technique for forming complex foldable structures. The initial substrate was formed of a three layer laminate, consisting of, in order, a stiff organic material for the panels (1 mm thick wood veneer, in this case), a thin Al foil to act as a blocking layer, and then a thin paper or Mylar layer to realize flexible hinges. All three materials were commercially available; layers were bonded with 3D LSE sheet adhesive. The fabricated origami structure is shown in Figure 2g.

Fabrication was started with an initial crease pattern description containing mountain/valley fold direction specifications and the desired fold angle of each (all folds were designed to accommodate ±90° or ±180°); the crease pattern was then processed by *Tessellatica*,^[^ ^97^ ^]^ an open‐source *Mathematica* package for the design and processing of fold patterns, to transform the crease pattern into a set of instructions for a laser cutter, incorporating kerfs as needed to allow for the desired range of folding motion.

The substrate was loaded into a laser cutter (YueMing Laser CMA960) and processed according to the transformed instructions. The power level was set high enough to burn through the substrate; however, at 10.6~μm wavelength, the Al foil barrier layer was highly reflective, and so the paper or Mylar layer underneath was unharmed. This resulted in a monolithic array of rigid panels separated by flexible hinges with sufficient kerfs to allow the desired range of motion.

### Metasurface Antenna Near‐Field Scans and Imaging Experiments

The metasurface antenna was mounted on a custom 3D printed antenna holder. The holder was placed on a custom build xy scanner stage controlled by an Arduino board. Port 1 of the VNA was connected to the antenna under test and port 2 to the coaxial to WR‐42 waveguide adapter that in turn was connected to an open‐ended waveguide near field probe. The aperture of the waveguide was exposed to and pointed to the antenna and the rest of the structure was covered by microwave absorber piece to minimize extraneous reflections (not shown). The maximum scan angle used in the setup was about ≈ 45°. This ensured most of the radiated fields to be captured by the probe. This was verified by comparing the simulated and measured vector fields. The near field scan setup is shown in Figure 3f. The cables and the switch matrix networks were calibrated out using the VNA. The RF switch matrix was used to switch and sweep between transmitter and receiver antennas. The RF switch network consisted of six single‐pole‐six‐throw (SP6T) electromechanical switch that required a 24 V control voltage. The Arduino board controlled these switch matrix. The further details are provided in Supporting Information and are explained in Figure S12, Supporting Information. The imaging experiments were performed inside a custom‐built anechoic chamber again to minimize extraneous reflections from the surroundings.

## Conflict of Interest

The authors declare no conflict of interest.

## Author Contributions

S.V. conceived the idea of computational imaging with metasurface antennas on origami platform. R.J.L. conceived the idea of grafted Waterbomb origami structure and built the origami platform. S.V. designed the metasurface antennas and X.L. designed the layout for fabrication. S.V., D.S., and X.L. built the imaging setup and performed all the measurements. S.V. performed the mathematical analysis, image reconstructions, and analyzed the experimental results. K.S. supervised the project. S.V. and R.J.L wrote the manuscript and all the authors provided their comments.

## Supporting information

Supporting Information

Supplemental Video 1

Supplemental Video 2

## Data Availability

The data that support the findings of this study are available from the corresponding author upon reasonable request.
