# Supplementary material for: Origami Microwave Imaging Array: Metasurface Tiles on a Shape‐Morphing Surface for Reconfigurable Computational Imaging
Source: Adv Sci (Weinh). 2022 Jul 27;9(28):2105016. doi: 10.1002/advs.202105016 (PMC9534976; doi:10.1002/advs.202105016)
Supplement: Supplementary file 1 — Supporting Information [file ADVS-9-2105016-s003.pdf]

ADVANCED SCIENCE
Open Access

Supporting Information

for *Adv. Sci.*, DOI 10.1002/advs.202105016

Origami Microwave Imaging Array: Metasurface Tiles on a Shape-Morphing Surface for Reconfigurable Computational Imaging

Suresh Venkatesh, Daniel Sturm, Xuyang Lu, Robert J. Lang and Kaushik Sengupta*

WILEY-VCH

Supplementary Material- Origami Microwave Imaging Array: Active Metasurface Tiles on a Shape-morphing Surface for Reconfigurable Computational Imaging

S. Venkatesh, et.al.

1 Line Source Excitation and Cylindrical Harmonic Decomposition

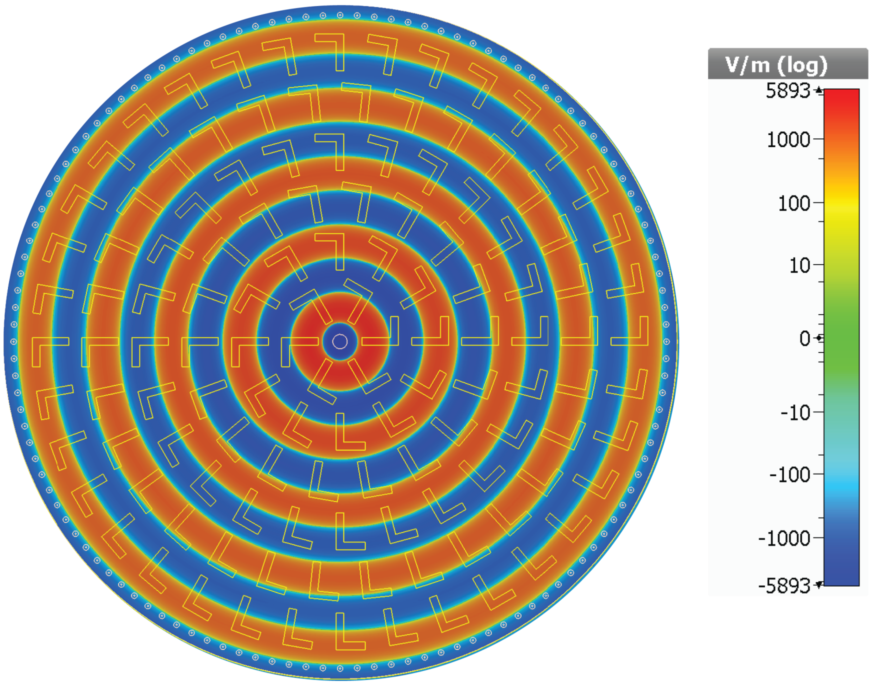

Figure S1: The real part of the $\mathbf{E}_z$ component of the excited linesource inside the metasurface cavity.

The coaxial cable inside the two parallel plate cavity filled with a dielectric excites a cylindrical line source which can be represented of the form,

$$\mathbf{E}_z\left(\rho,\phi\right) = -\frac{\beta^2 I_0}{4\omega\epsilon_0\epsilon_r} H_0^{(2)}\left(\beta_\rho\rho\right) \tag{1}$$

where, I_0 is the electric current, ϵ_r is the effective dielectric constant of the cavity, $\beta = \sqrt{\beta_\rho^2 + \beta_z^2}$, and $H_0^{(2)}$ is the Hankel function of second kind.
This excited field is scattered due to the slot array structure. The scattered transverse fields at a plane z with a radius a, can be decomposed into cylindrical harmonics as follows,
Transverse magnetic (TM) field modes

$$\mathbf{E}_{mn}^{TM} = \left[E_{\rho mn}^{TM}(\rho,\phi)\hat{\boldsymbol{\rho}} + E_{\phi mn}^{TM}(\rho,\phi)\hat{\boldsymbol{\phi}} + E_0 J_m(\beta_{mn}\rho)e^{-jm\phi}\hat{\mathbf{z}}\right] e^{-j\beta_{zmn}z} \tag{2}$$

$$\mathbf{H}_{mn}^{TM} = \left[H_{\rho mn}^{TM}(\rho,\phi)\hat{\boldsymbol{\rho}} + H_{\phi mn}^{TM}(\rho,\phi)\hat{\boldsymbol{\phi}}\right] e^{-j\beta_{zmn}z} \tag{3}$$

Transverse electric (TE) field modes

$$\mathbf{E}_{mn}^{TE} = \left[E_{\rho mn}^{TE}(\rho,\phi)\hat{\boldsymbol{\rho}} + E_{\phi mn}^{TE}(\rho,\phi)\hat{\boldsymbol{\phi}}\right] e^{-j\beta'_{zmn}z} \tag{4}$$

$$\mathbf{H}_{mn}^{TE} = \left[H_{\rho mn}^{TE}(\rho,\phi)\hat{\boldsymbol{\rho}} + H_{\phi mn}^{TE}(\rho,\phi)\hat{\boldsymbol{\phi}} + \frac{E_0}{\eta} J_m(\beta'_{mn}\rho)e^{-jm\phi}\hat{\mathbf{z}}\right] e^{-j\beta'_{zmn}z} \tag{5}$$

Radial and axial propagation constants aare given by,

$$\beta_{mn} = \frac{\chi_{mn}}{a} \tag{6}$$

$$\beta'_{mn} = \frac{\chi'_{mn}}{a} \tag{7}$$

$$\beta_{zmn} = \sqrt{\beta^2 - \left(\frac{\chi_{mn}}{a}\right)^2} \tag{8}$$

$$\beta'_{zmn} = \sqrt{\beta^2 - \left(\frac{\chi'_{mn}}{a}\right)^2} \tag{9}$$

where χ_{mn} and χ'_{mn} represents the nth zero of bessel function J_m and bessel derivative function J'_m (of first kind of order m), respectively.
Defining dimensionless transverse radial basis functions,

$$E_0 \boldsymbol{\psi}^{TM}_{mn}(\rho)e^{-jm\phi} = E^{TM}_{\rho mn}(\rho,\phi)\hat{\boldsymbol{\rho}} + E^{TM}_{\phi mn}(\rho,\phi)\hat{\boldsymbol{\phi}} \tag{10}$$

$$E_0 \boldsymbol{\psi}^{TE}_{mn}(\rho)e^{-jm\phi} = E^{TE}_{\rho mn}(\rho,\phi)\hat{\boldsymbol{\rho}} + E^{TE}_{\phi mn}(\rho,\phi)\hat{\boldsymbol{\phi}} \tag{11}$$

Evaluating the basis functions,

$$\boldsymbol{\psi}^{TM}_{mn}(\rho) = \frac{j\beta_{zmn}}{2\beta_{mn}}\left[\left[J_{m+1}(\beta_{mn}\rho) - J_{m-1}(\beta_{mn}\rho)\right]\hat{\boldsymbol{\rho}} + \frac{2jm}{\beta_{mn}\rho}J_m(\beta_{mn}\rho)\hat{\boldsymbol{\phi}}\right] \tag{12}$$

$$\boldsymbol{\psi}^{TE}_{mn}(\rho) = \frac{j\beta}{2\beta'_{mn}}\left[\frac{2jm}{\beta'_{mn}\rho}J_m(\beta'_{mn}\rho)\hat{\boldsymbol{\rho}} + \left[J_{m-1}(\beta'_{mn}\rho) - J_{m+1}(\beta'_{mn}\rho)\right]\hat{\boldsymbol{\phi}}\right] \tag{13}$$

Field expansion in terms of TM and TE modes are given by,

$$\mathbf{E} = \sum_{m,n} \alpha^{TM}_{mn}\mathbf{E}^{TM}_{mn} + \alpha^{TE}_{mn}\mathbf{E}^{TE}_{mn} \tag{14}$$

$$\mathbf{H} = \sum_{m,n} \alpha^{TM}_{mn}\mathbf{H}^{TM}_{mn} + \alpha^{TE}_{mn}\mathbf{H}^{TE}_{mn} \tag{15}$$

where α^{TM}_{mn} and α^{TE}_{mn} are the TM and TE mode coefficients, respectively.
Transverse electric field expansion is given by,

$$\mathbf{E}_t(\rho,\phi,z) = E_0\sum_{m,n}\left[\alpha^{TM}_{mn}\boldsymbol{\psi}^{TM}_{mn}(\rho)e^{-j\beta_{zmn}z} + \alpha^{TE}_{mn}\boldsymbol{\psi}^{TE}_{mn}(\rho)e^{-j\beta'_{zmn}z}\right]e^{-jm\phi} \tag{16}$$

Given the near vector fields from the measured near-field scans, $\mathbf{E}$, these fields can be approximated and converted to magnetic surface currents using surface equivalence theorem,

$$\mathbf{M_s} = -2\hat{\mathbf{n}} \times \mathbf{E} \tag{17}$$

where, $\hat{\mathbf{n}} = \hat{\mathbf{z}}$ is the surface normal. The magnetic surface current in the aperture plane can be converted to a set of magnetic dipoles,

$$\mathbf{m}_p = \left(\frac{j\Delta x\Delta y}{\omega\mu_0}\right)\mathbf{M_s} \tag{18}$$

where, Δx and Δy are the near field pixel dimensions used for discretization and ω is the angular frequency. These individual dipoles can be propagated to the scene plane of interest using Green's function and summed to get the overall response at a given scene voxel as follows.

$$\mathbf{E}(\mathbf{r}_s;\mathbf{r}_a,\omega) = \frac{-j\omega\mu_0}{4\pi}\sum_p\left[\left(\frac{\mathbf{m}_p \times \mathbf{r}_S}{|\mathbf{r}_S|}\right)\left(\frac{-jk}{R_p} - \frac{1}{R_p^2}\right)\exp(-jkR_p)\right] \tag{19}$$

where, k=$2\pi/\lambda$ is the wavenumber, $R_p = |\mathbf{r}_s - \mathbf{r}_a|$, is the distance of a pth magnetic dipole to the scene voxel. Here $\mathbf{r}_s$ is the radial vector from the origin of the aperture to the voxel at the scene plane and $\mathbf{r}_a$ is the radial vector from the origin to the location of the point magnetic dipole in the aperture plane.

2 Metasurface Antenna Tile Characterization

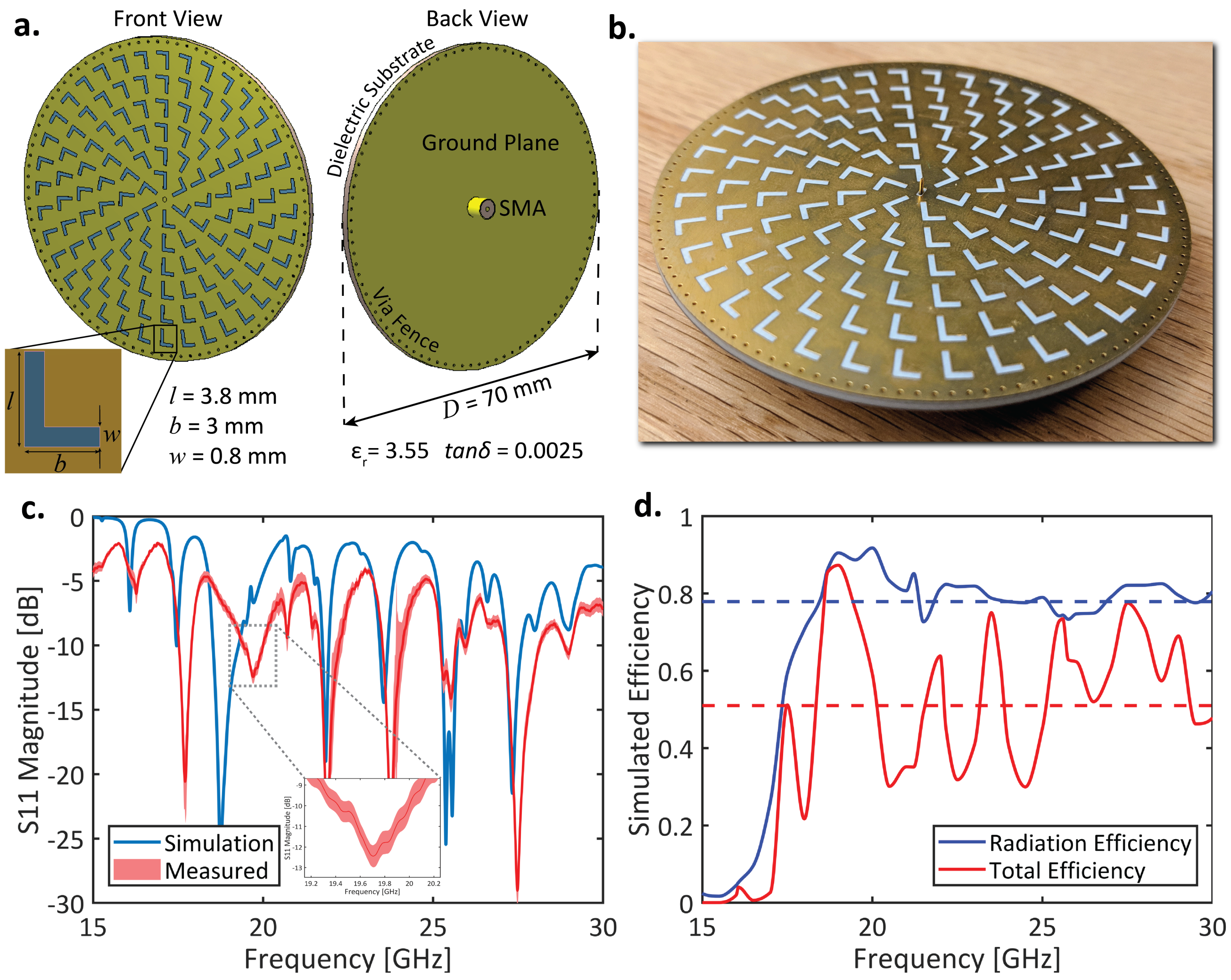

Figure S2: Metasurface antenna design and characterization. **a.** The metasurface antenna with subwavelength 'L' shaped slots repeated radially on the top metal surface. The bottom plane is the ground plane with a dielectric separation and the antenna is fed in the center with a coaxial feed. **b.** The fabricated metasurface antenna is shown. **c.** Simulated and measured S_{11} as a function of frequency showing the broadband characteristics of the metasurface antenna from 17-27 GHz. The variation in S_{11} for all the 22 antennas used in the setup is also plotted and is also highlighted in the zoomed plot. **d.** The simulated radiation and total efficiency of the metasurface antenna.

Fig. S2a shows the design dimensions of the Ka-band 17 - 27 GHz metasurface antenna. The antenna was designed on a Rogers 4003 substrate with a dielectric constant, $\epsilon_r = 3.55$ and a loss tangent, $\tan\delta = 0.0025$. Overall diameter of the antenna is about 70 mm. The top side of the antenna is patterned with 'L' shaped radial slots. The fabricated antenna is shown in Fig. S2b. The simulated and measured absolute reflection coefficients (S_{11} magnitude) are shown in Fig S2c. The S_{11} measurements are repeated for all the 22 antennas used in the setup and all the antennas exhibit similar performance with an overall variation in S_{11} of approximately ± 0.5 dB across the operating band. The antenna radiates with average radiation and total efficiency of 80% and 50%, respectively, as shown in Fig. S2d. The antenna is excited with a coaxial feed at the bottom which excites a uniform line source inside the cylindrical cavity. The excited field then radiates out through the radial slot on the top surface leading to frequency diverse nature of the antenna patterns.The measurement variation of S_{11} magnitude across the 22 antenna elements include all the cabling to complex switch network (shown in Fig. S12). We ensure that the S-parameters of the switch matrix network is very well embedded and calibrated across the array. This in turn helps in avoiding additional calibration while constructing the image transfer matrix, **H**.

3 Laguerre-Gaussian Orbital Angular Momentum Beam Evolution

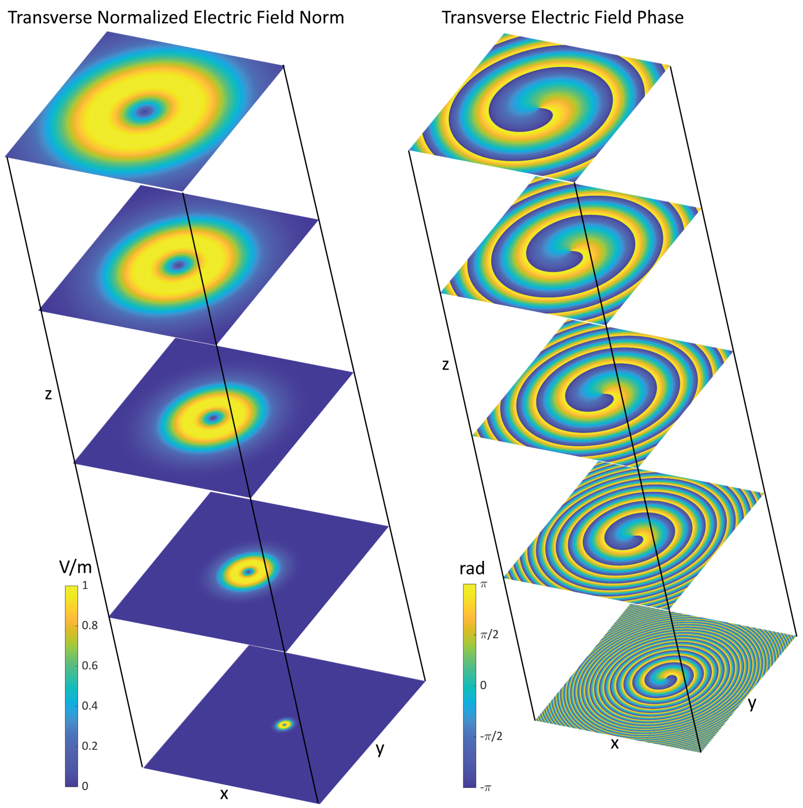

Figure S3: Evolution of $\mathcal{L}_1^{|-1|}$ Laguerre-Gaussian Orbital Angular Momentum Beam

Figure. S3 shows the evolution of $\mathcal{L}_1^{|-1|}$ mode Laguerre-Gaussian orbital angular momentum beam along the z direction. The beam phase profile resembles that of the metasurface in the lower and mid-band frequency ranges. However, at high-band the metasurface radiates higher order modes and hence deviates from $\mathcal{L}_1^{|-1|}$ OAM mode.

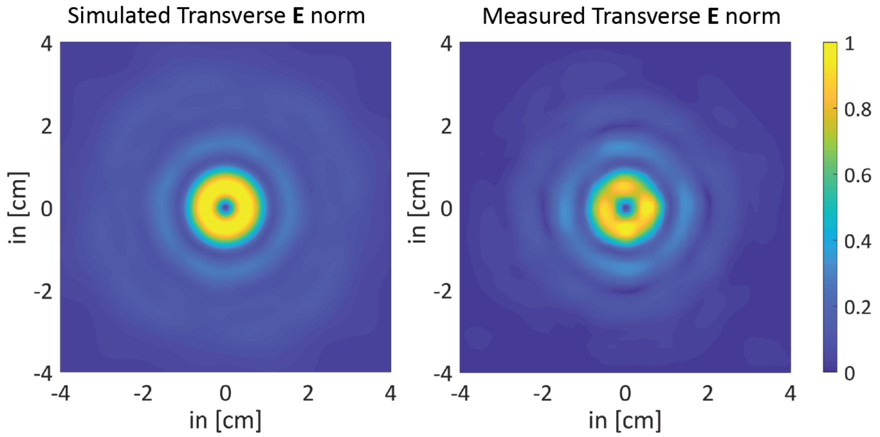

Figure S4: Metasurface antenna simulated and measured normalized transverse electric field norm (near-field) at 17 GHz.

Figure. S4 shows the simulated and measured normalized transverse electric field norm of the broadband metasurface antenna at 17 GHz. Transverse electric field norm $\mathbf{E}_t = \sqrt{\mathbf{E}_x^2 + \mathbf{E}_y^2}$. Due to the symmetric structure of the metasurface antenna and the axially symmetric excitation from the SMA probe, $\mathbf{E}_x$ and $\mathbf{E}_y$ are orthogonal to each other as mentioned in Fig. 3.

4 Comparison of the 3D target reconstruction with ground truth

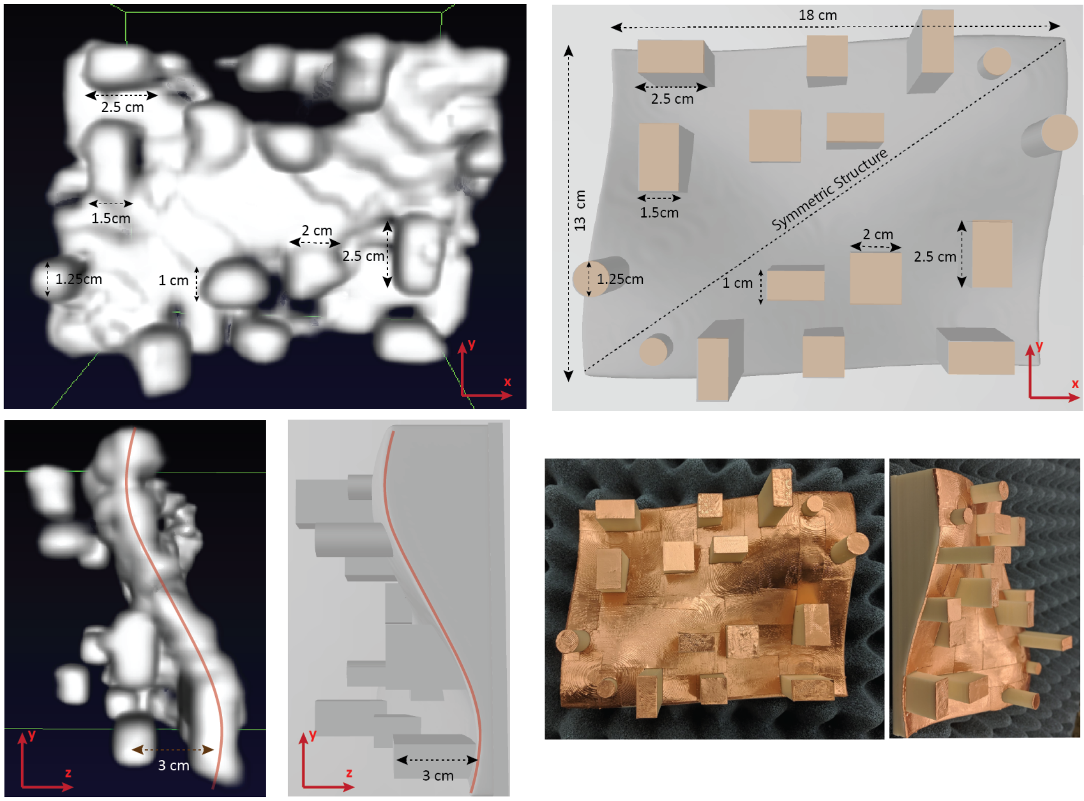

Figure S5: Comparison of the reconstructed complex 3D target using the curved origami with the ground truth.

The above Fig. S5 shows the image reconstruction of the complicated 3D printed target using the curved origami (structure shown in Fig. 5.a). The reconstructed image is compared with the ground truth. The 3D target is symmetric about the diagonal with undulating surface and variable projection stubs sticking out of the surface which help to characterize both the complex specular reflections and depth resolution of the reconstructed image simultaneously.

5 Cylindrical target reconstruction with curved and planar origami platforms

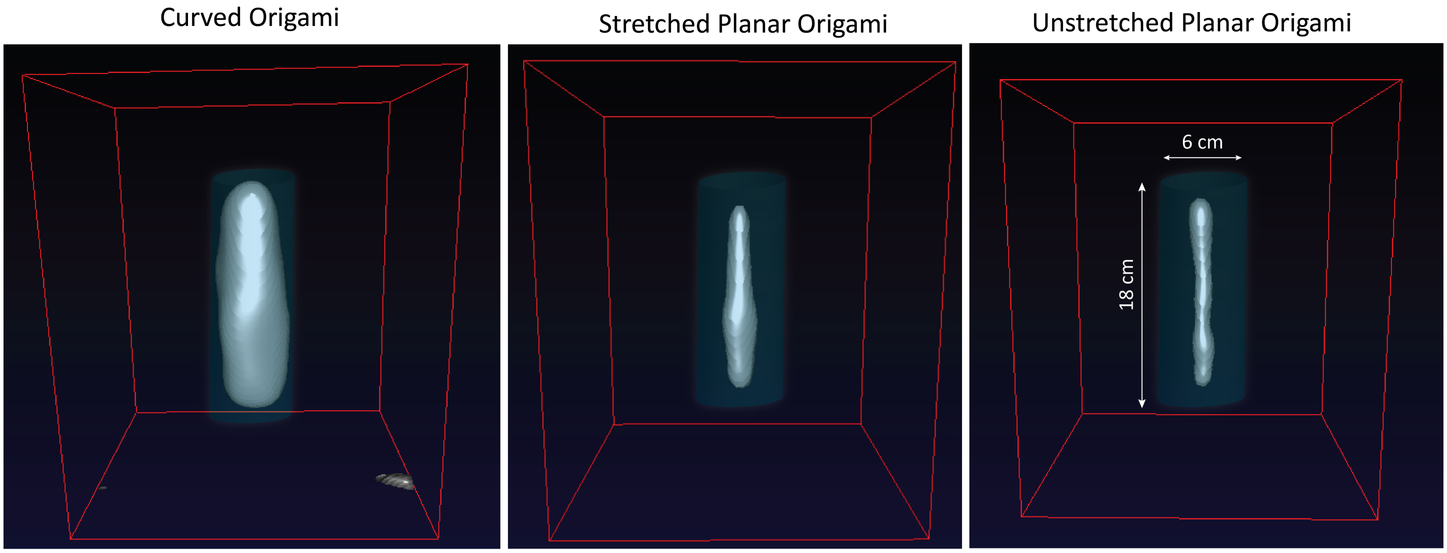

Figure S6: Image reconstruction at a standoff distance of 1m for a simple, specular cylindrical scattering structure with curved and planar origami platforms (including both stretched and unstretched cases).

The above Fig. S6 shows the reconstruction comparison between curved (structure shown in Fig. 5.a) and planar origami (both stretched and unstretched planar origami platform shown in Fig .4a and b respectively) of a convex shaped cylindrical target at 1 m standoff. The cylindrical target is 6 cm in diameter and 18 cm in height. The figure shows the ability of curved origami to reconstruct specular target effectively. The reconstruction uses 11 transmitter and 11 receiver metasurface antennas with a total number of 101 frequency steps from 17 to 27 GHz leading to a total number of measurements of 12221.

6 Stereographic Camera for Mapping the Origami Structure.

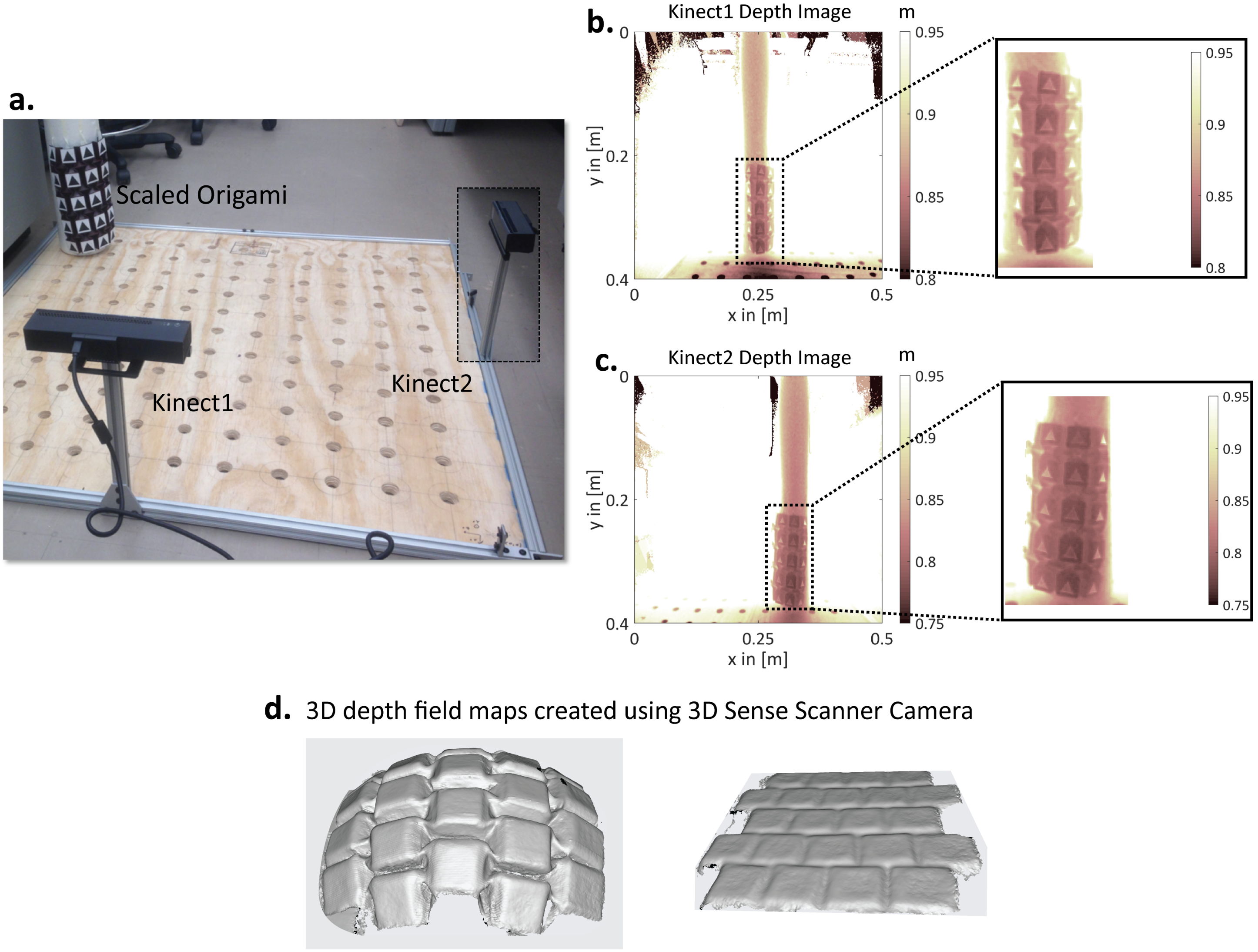

Figure S7: Mapping 3D surfaces using stereographic depth field cameras.

The construction of image transfer matrix **H** requires the exact location of Tx and Rx locations and their corresponding near field maps. The locations of the Tx and Rx tiles is determined using a commercial off-of-the-shelf stereographic camera, 3D sense scanner to map the 3D origami structure. The 3D structure can be imported into any computer-aided design (CAD) software to determine the center locations of the antenna tiles. The accuracy of the these depth field images is about ±5 mm. This technique is also verified using two orthogonally placed Microsoft Kinect cameras.

7 3D Target Imaging Setup

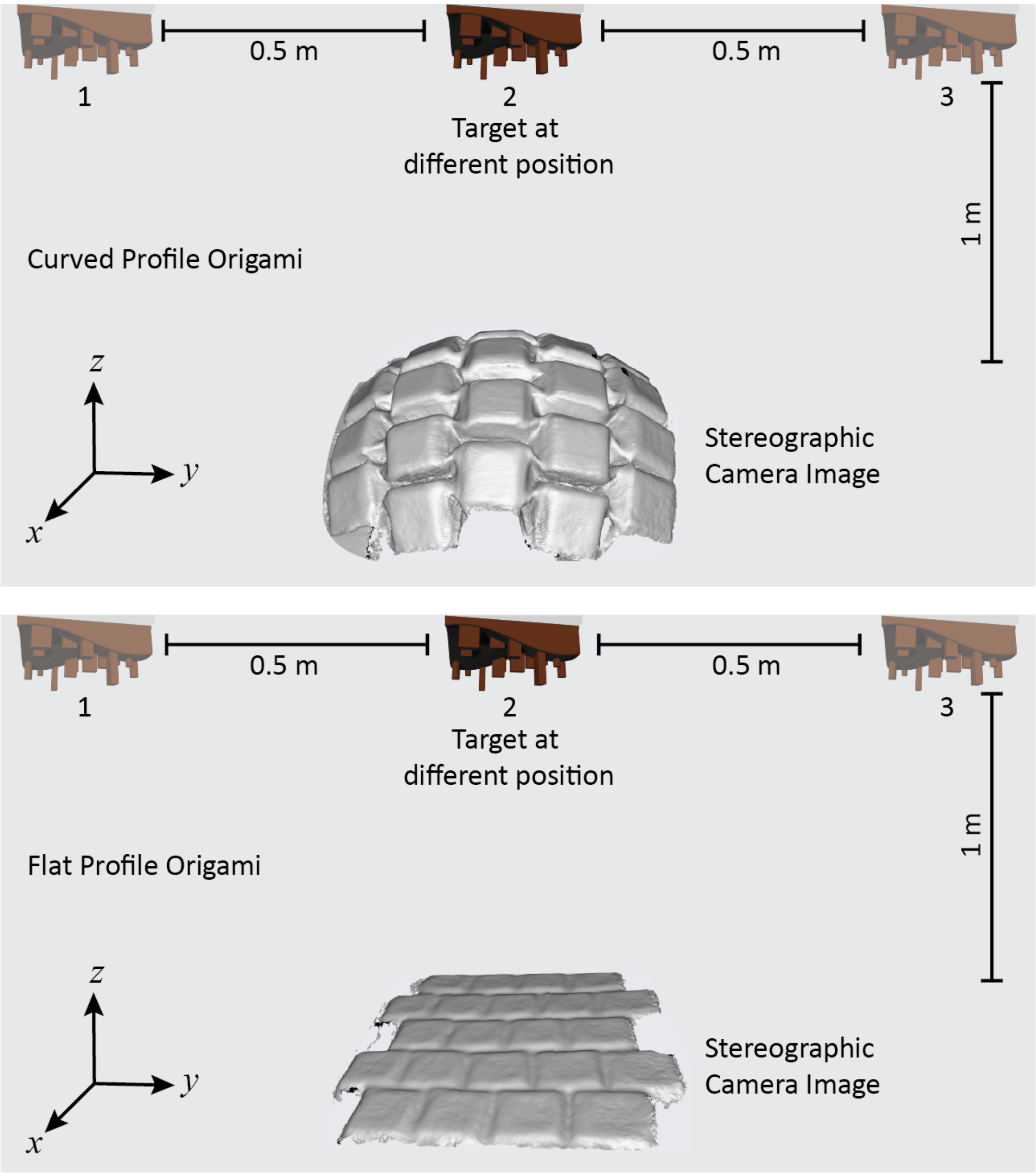

Figure S8: 3D target imaging experiment setup showing the ability to co-add images from different positions.

3D image reconstructions shown in Fig. 5 are constructed by co-adding three offset images from the center of the origami aperture. Firstly, the measurements are performed and image is reconstructed with the target object at the center and later two more reconstructions are performed with the target object offset from the center as shown in Fig S8. These three images are co-added with respect to a custom reference point to construct the final image. The curved origami structure demonstrates better field-of-view compared to its flat counterpart.

8 Antenna Location Error Effects on Image Reconstructions

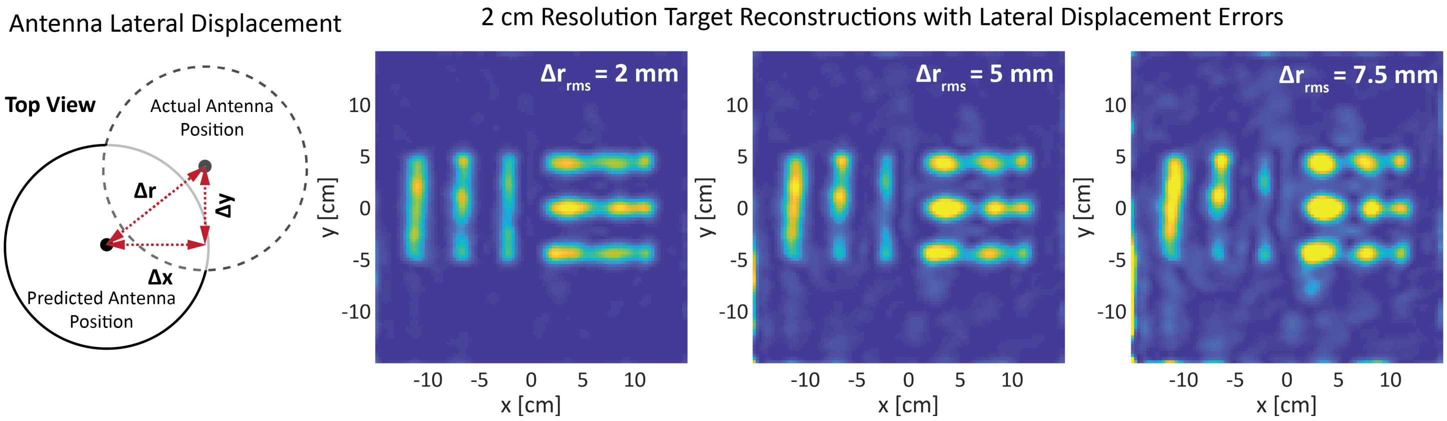

Figure S9: Effect of lateral antenna displacement error on image reconstructions.

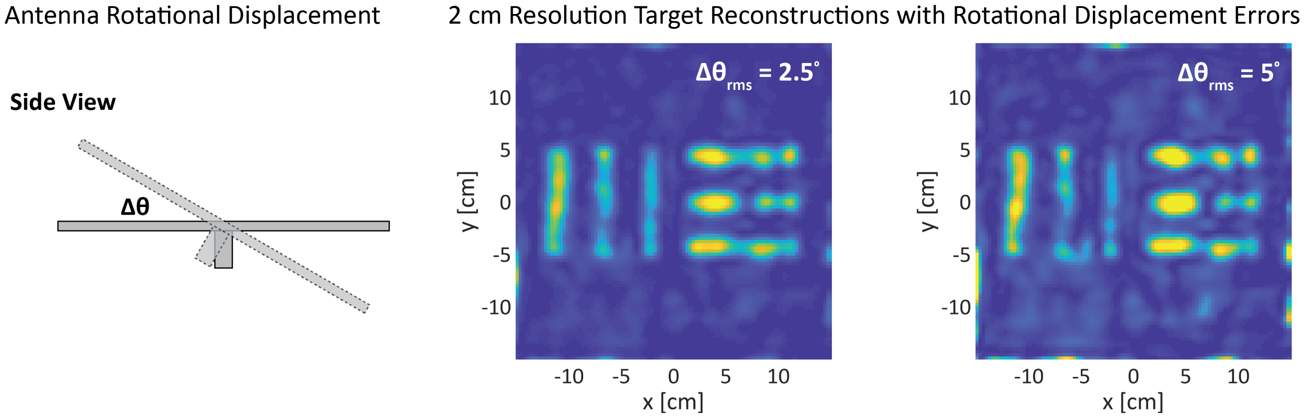

Figure S10: Effect of antenna rotational displacement error on image reconstructions.

Figures. S9 and S10 show the effect of varied antenna lateral and rotational displacement on 2 cm resolution target image reconstructions. The current image reconstruction method and system can potentially tolerate a lateral rms error, $\Delta r_{rms} = 7.5$ mm and rotational rms error, $\Delta\theta_{rms} = 5°$. The accuracy of determining the antenna positions in 3D space is limited by the point cloud data acquired from the stereographic camera whose depth accuracy is 5 mm and cross range accuracy is 1 mm within the working range and field of view. The above simulations errors are higher than the quoted accuracy of the stereographic camera. Hence this leads to high fidelity image reconstructions shown in the main manuscript in Fig. 4 and 5. Suppose the misalignment of the antenna positions are known, there are matrix-based techniques to adaptively correct the image reconstructions [89, 90].

9 Metasurface: Via cage analysis

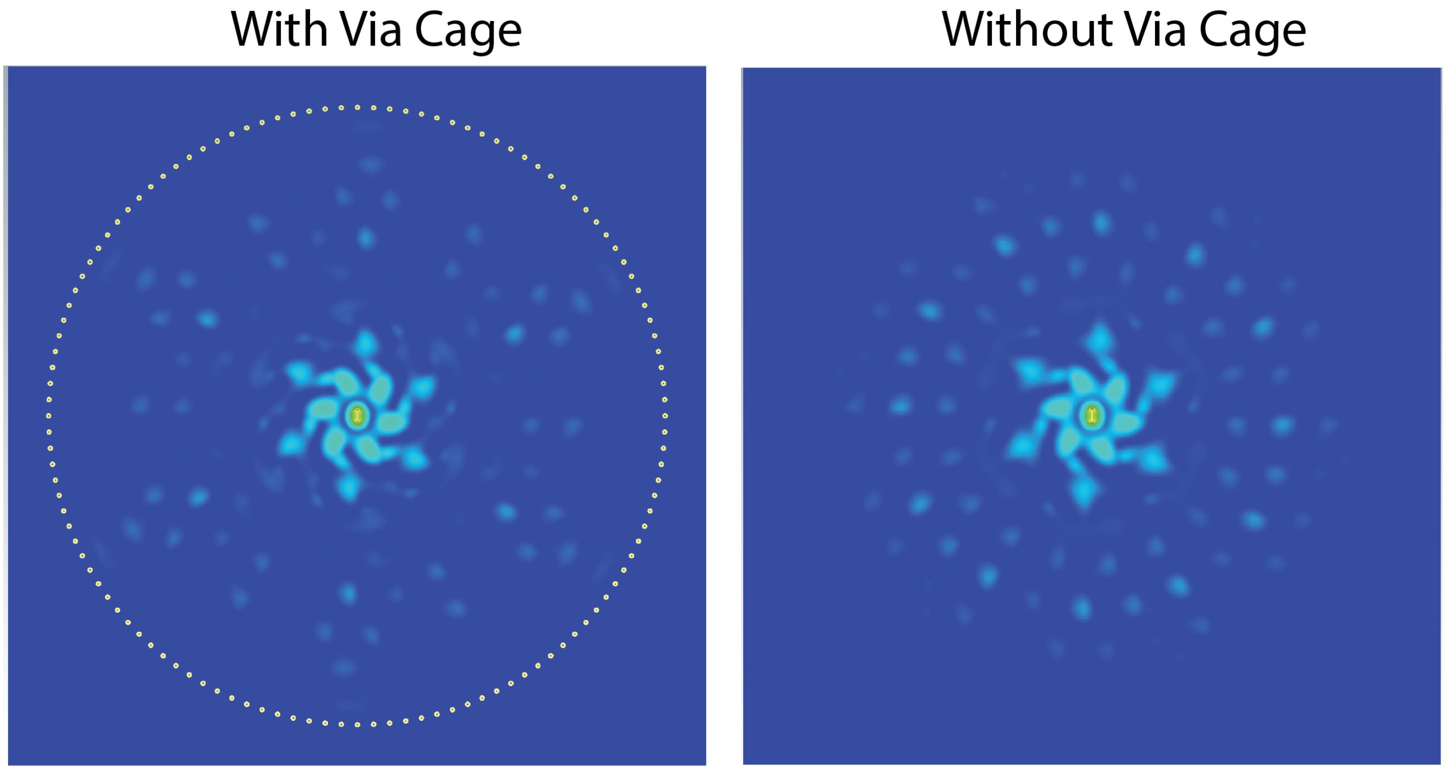

Figure S11: Near field electric norm at 21 GHz with and without via cage.

Figure. S11 shows the near field electric norm at 21 GHz with and without via cage. Via cage helps in minimizing the effects of mutual coupling between the antennas when oriented in different angles including planar configurations. The response of the antenna mostly remains the similar with and without the via cage as most of the guide mode is leaked out through the top layer slots before it reaches the edge of the antenna.

10 Switch Matrix for Transmitter and Receiver pair Selection

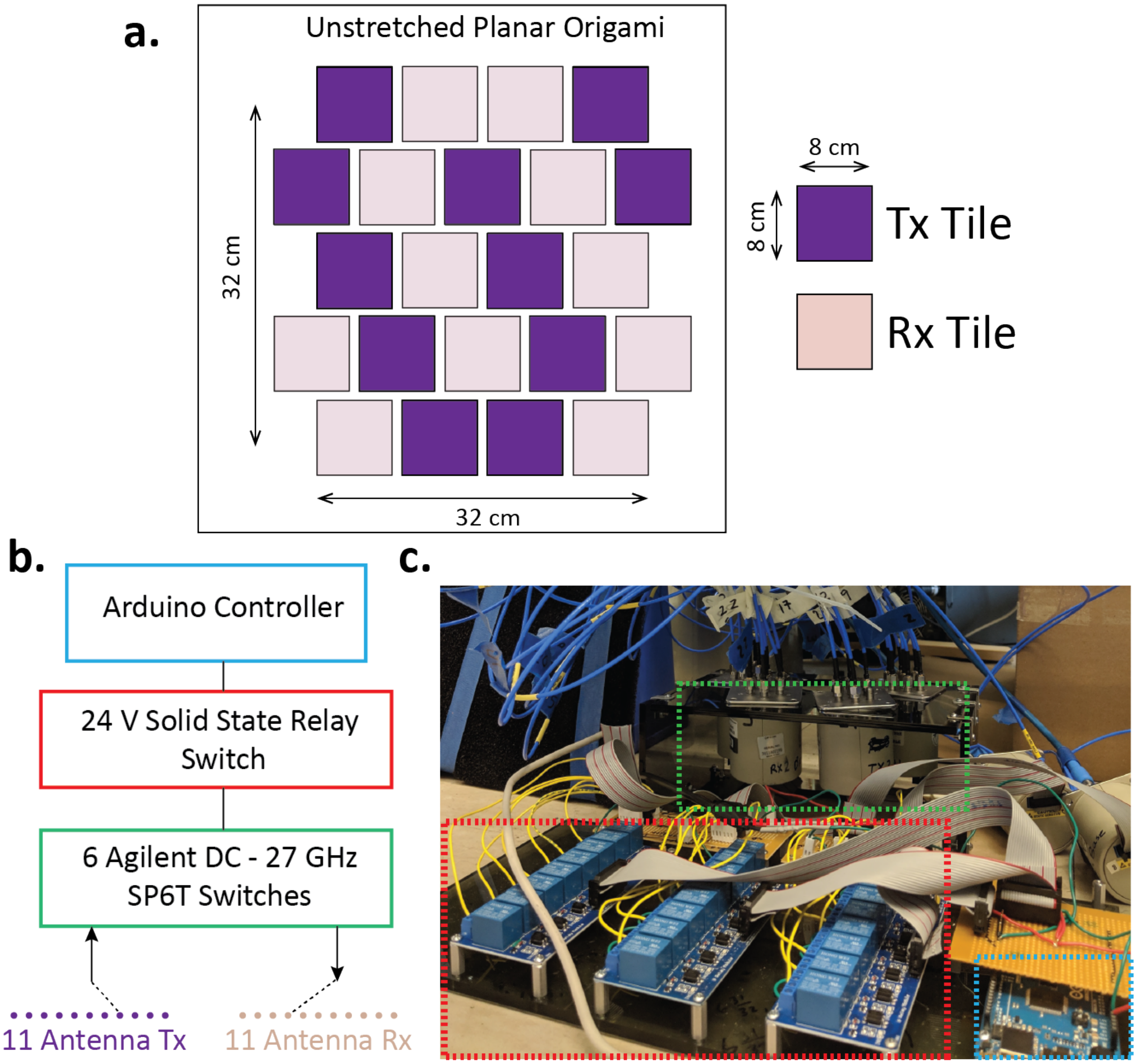

Figure S12: Switch matrix architecture to select the different transmitter and receiver pairs from 22 antenna tile sets.

Initial transmitter and receiver antenna tile set are pre-selected and is shown in Fig S12 a. This set is chosen in a way to minimize redundant symmetric combination and to maintain good spatial diversity.This set can also be chosen by **H** matrix optimization. The switch matrix consists of 6 single pole six throw (SP6T) RF electro-mechanical switches (DC - 27 GHz). These 6 SP6T switches are then controlled externally through a solid state switch which is again controlled through a programmable Arduino controller. The switch matrix system overview and the setup are shown in Fig. S12 b and c, respectively.
A part of the switch matrix selects a Tx antenna 11 sets pre-selected antennas and connects to Port 1 of the VNA and similarly another part of switch matrix selects from the remaining 11 antennas to connect a particular Rx antenna to Port 2 of the VNA. The custom MATLAB program controls the Ardunio board which selects pair 121 pairs of Tx and Rx combinations. After each combination is selected, the MATLAB program then controls the Vector Network Analyzer (VNA) through a GPIB cable to record the transmission coefficients (S21) across frequency.
